# Single‐Cell and Machine Learning Analyses Identify MYDGF as an Immune‐Related Biomarker Associated With the Tumor Microenvironment in Clear Cell Renal Cell Carcinoma

**DOI:** 10.1155/humu/5262666

**Published:** 2026-07-25

**Authors:** Yingkun Xu, Guandu Li, Xinxiu Ren, Xiaochen Qi, Guangzhen Wu

**Affiliations:** ^1^ Department of General Surgery, Qilu Hospital of Shandong University, Jinan, China, qiluhospital.com; ^2^ Department of Urology, The First Affiliated Hospital of Dalian Medical University, Dalian, China, dlmedu.edu.cn; ^3^ Department of Cell Biology, College of Basic Medical Science, Dalian Medical University, Dalian, China, dlmedu.edu.cn

**Keywords:** clear cell renal cell carcinoma, immune infiltration, machine learning, MYDGF, prognosis, single-cell RNA sequencing, tumor microenvironment

## Abstract

Single‐cell transcriptomics and machine learning methods are increasingly used to identify immune‐related biomarkers in solid tumors, yet their combined application to microenvironment‐related drivers of therapeutic resistance in clear cell renal cell carcinoma (ccRCC) is still limited. Here, we investigated the biological and clinical significance of myeloid‐derived growth factor (MYDGF) through an integrative strategy spanning single‐cell profiling, bulk multiomics, and functional validation. Analysis of scRNA‐seq data (GSE156632) revealed that MYDGF is preferentially detected in malignant epithelial subpopulations and associated with the composition of myeloid and lymphoid compartments. Integration with TCGA‐KIRC transcriptomic and clinical datasets demonstrated strong associations between MYDGF expression and immune‐checkpoint activation, immune dysfunction signatures, and PI3K/AKT–MAPK pathway activity. Tumors with high MYDGF expression exhibited an immune‐infiltrated yet functionally impaired microenvironment and were predicted to show reduced responsiveness to immune checkpoint blockade. Differential expression and enrichment analyses further highlighted MYDGF‐associated genes involved in inflammatory, extracellular, and receptor‐binding functions. A machine learning pipeline using LASSO Cox regression identified a preliminary 19‐gene MYDGF‐related prognostic gene set that requires further validation. Functional experiments confirmed that MYDGF knockdown suppressed proliferation, migration, and invasion in ccRCC cells. Overall, our analyses characterize MYDGF as a microenvironment‐related biomarker linked to immune‐associated features, signaling‐associated alterations, and adverse prognosis in ccRCC. These results nominate MYDGF as a candidate prognostic biomarker and show the value of pairing single‐cell resolution with computational modeling for biomarker discovery in renal cancer.

## 1. Introduction

Renal cancer represents the second most common and lethal malignancy of the urinary system worldwide. Among its histological subtypes, clear cell renal cell carcinoma (ccRCC) accounts for more than 75% of cases. Approximately 30% of patients present with distant metastases at the time of diagnosis, and the 5‐year survival rate in this population remains below 10% [1]. Although targeted therapies, such as vascular endothelial growth factor (VEGF) inhibitors and immune checkpoint inhibitors, including PD‐1/PD‐L1 antibodies, have substantially improved outcomes for patients with advanced ccRCC, nearly half of these patients ultimately experience treatment failure due to primary resistance or disease recurrence [1–3]. This limited therapeutic benefit largely reflects the pronounced intertumoral and intratumoral heterogeneity characteristic of ccRCC [4, 5]. Consequently, elucidating the cellular and molecular mechanisms underlying ccRCC progression and identifying novel therapeutic targets remain critical for improving patient outcomes.

The tumor microenvironment (TME) plays a central role in driving ccRCC progression and therapeutic resistance [6]. The ccRCC microenvironment is characterized by abundant immunosuppressive cell populations, such as M2‐polarized macrophages and regulatory T cells, as well as multiple protumorigenic factors [5, 7]. Together, these components contribute to malignant progression by modulating tumor cell proliferation, angiogenesis, and immune evasion. Accumulating evidence suggests that tumor growth and progression are closely associated with aberrantly expressed genes [3]. Based on this premise, we compared gene expression profiles between tumor and adjacent nontumor tissues and identified myeloid‐derived growth factor (MYDGF) as a candidate gene of particular interest.

MYDGF was originally identified in the context of skeletal muscle repair. The MYDGF gene is located on Chromosome 4q31.21 and has been reported to exert cytoprotective, proangiogenic, and anti‐inflammatory effects through the activation of signaling pathways such as PI3K/Akt and MAPK [8]. Recent studies have demonstrated that MYDGF is highly expressed in hepatocellular carcinoma and lung cancer, where its expression correlates with tumor size, metastatic potential, and poor clinical outcomes. In hepatocellular carcinoma, MYDGF has been shown to promote sorafenib resistance by inducing transcriptional activation of ANGPT2 [9], whereas in lung cancer it has been identified as an oncogenic factor [10]. However, the expression pattern and functional role of MYDGF in ccRCC have not yet been elucidated.

Conventional bulk RNA sequencing approaches are limited in their ability to resolve gene expression differences between tumor cells and cells within the TME. In contrast, single‐cell RNA sequencing (scRNA‐seq) enables transcriptomic profiling at single‐cell resolution, allowing precise identification of the cellular sources of functionally relevant genes. As demonstrated in previous integrative studies, combining scRNA‐seq with bulk transcriptomics provides a robust framework for disentangling cell type–specific contributions to tumor heterogeneity and therapeutic resistance [11, 12].

Recent advances in single‐cell sequencing coupled with machine learning approaches have enabled high‐resolution characterization of intratumoral heterogeneity and immune microenvironment dynamics. These integrative strategies facilitate the identification of clinically meaningful biomarkers that may be overlooked by conventional bulk analyses. However, such combined approaches have rarely been applied to ccRCC, leaving the contribution of specific microenvironment‐associated genes insufficiently understood.

In this study, we analyzed the scRNA‐seq dataset GSE156632 from the GEO database, which includes seven ccRCC samples and five normal renal samples, in combination with TCGA‐KIRC multiomics data and in vitro experimental validation. Through this integrated approach, we are aimed at elucidating the role of MYDGF in ccRCC and at providing experimental evidence supporting its potential utility as a diagnostic biomarker and therapeutic target.

## 2. Methods

### 2.1. Data Collection

scRNA‐seq data for renal cell carcinoma were obtained from the Gene Expression Omnibus (GEO) database (https://www.ncbi.nlm.nih.gov/geo/). The dataset GSE156632 includes a total of 12 samples, comprising seven ccRCC samples and five normal renal tissue samples. Bulk RNA sequencing data and corresponding clinical information were retrieved from The Cancer Genome Atlas (TCGA) database, specifically from the TCGA‐KIRC cohort, which consists of 539 tumor samples and 72 normal renal tissue samples.

### 2.2. scRNA‐Seq Data Analysis

Single‐cell transcriptomic data were processed using the Seurat R package (Version 4.1.0) [13]. Quality control was first performed to retain high‐quality cells for downstream analyses. Genes expressed in fewer than three cells were excluded, and cells expressing fewer than 200 genes were removed. Cells with mitochondrial gene content exceeding 20% were filtered out. In addition, cells with unique molecular identifier (UMI) counts greater than 1000 and with detected gene numbers ranging from 200 to 8000 were retained for further analysis.

Data normalization was performed using the LogNormalize method, and the Top 2000 highly variable genes were identified using the FindVariableFeatures function. The data were subsequently scaled using ScaleData. Principal component analysis (PCA) was conducted on the highly variable genes using the RunPCA function to capture the major sources of variation. To correct for batch effects across different samples, the Harmony algorithm was applied for data integration [14].

Dimensionality reduction and clustering were performed using UMAP and t‐SNE algorithms. The number of principal components used for clustering was determined based on ElbowPlot analysis. Using the first 15 principal components, FindNeighbors was applied to construct the shared nearest neighbor graph, and FindClusters was used for clustering across a range of resolutions (0.01–2). Clustree analysis was employed to visualize cluster stability across different resolutions, and a resolution of 1.5 was selected for downstream analyses, yielding a total of 42 cell clusters.

Marker genes for each cluster were identified using the FindAllMarkers function. Cell‐type annotation was performed based on canonical marker genes visualized by DotPlot, in combination with SingleR‐assisted annotation and manual curation [15]. Nine cell populations were initially annotated, including epithelial cells, endothelial cells, fibroblast cells, macrophages, monocytes, dendritic cells, T cells, NK cells, and B cells. For downstream interpretation and consistency across the manuscript, these populations were further summarized into six major lineages: epithelial/tumor cells, endothelial cells, myeloid cells, T/NK cells, B cells, and stromal cells. Specifically, macrophages, monocytes, and dendritic cells were grouped as myeloid cells; T cells and NK cells were grouped as T/NK cells; fibroblast cells were grouped as stromal cells; and epithelial cells were considered the epithelial/tumor compartment. The same six‐lineage nomenclature is used consistently throughout the Methods, Results, and figure legends.

To distinguish malignant epithelial cells from normal epithelial cells within the epithelial/tumor compartment, single‐cell copy‐number variation (CNV) profiles were inferred using the copyKAT algorithm with default parameters; epithelial cells classified as aneuploid were designated malignant tumor cells, whereas diploid epithelial cells were considered normal epithelial cells. Candidate genes were then screened by intersecting three criteria: (i) genes upregulated in malignant versus normal epithelial cells (|log2 fold change | [log2FC] > 0.585, false discovery rate < 0.05); (ii) genes significantly upregulated in tumor versus normal tissues in the TCGA‐KIRC cohort; and (iii) genes significantly associated with worse overall survival (OS) in univariate Cox analysis, yielding 30 candidate genes for downstream analyses. Cluster annotations were mapped onto UMAP and t‐SNE plots for visualization.

### 2.3. Identification and Functional Enrichment Analysis of Differentially Expressed Genes (DEGs)

DEGs between myeloid‐derived growth factor high‐expression (MYDGF‐H) and myeloid‐derived growth factor low‐expression (MYDGF‐L) groups were identified using the “limma” R package. Genes with an absolute log2FC > 1 and *p* < 0.05 were considered statistically significant. A total of 464 upregulated and 207 downregulated DEGs were identified. Heatmaps were generated using the “pheatmap” package, and gene annotation was performed with the “http://org.Hs.eg/.db” package. Gene Ontology (GO) and Kyoto Encyclopedia of Genes and Genomes (KEGG) pathway enrichment analyses were conducted using the “clusterProfiler” package.

### 2.4. Prognostic Analysis

Patients were stratified into MYDGF‐H and MYDGF‐L groups based on the optimal cutoff value. The optimal cutoff value of MYDGF expression was determined using the surv_cutpoint function of the survminer R package, which selects the expression threshold yielding the most significant separation of survival outcomes. Kaplan–Meier survival analyses were performed to evaluate the association between MYDGF expression and clinical outcomes. Univariate and multivariate Cox proportional hazards regression analyses were conducted to assess the prognostic significance of MYDGF in combination with clinical variables. Survival curves were generated using the “survival” R package.

### 2.5. Immune Infiltration Analysis

Immune cell infiltration was estimated using multiple computational algorithms, including TIMER [16], CIBERSORT [17], CIBERSORT‐ABS [17], quanTIseq [18], MCP‐counter [19], xCell [20], and EPIC [21]. In addition, the Tumor Immune Dysfunction and Exclusion (TIDE) algorithm was applied to predict patient responses to immunotherapy [22].

### 2.6. Genetic Characteristics Analysis

Tumor mutational burden (TMB) and microsatellite instability (MSI) scores were obtained from the TCGA database. Somatic mutation profiles of MYDGF in TCGA‐KIRC patients were extracted and visualized. The mRNA expression‐based stemness index (mRNAsi) was derived from a previously published study based on the one‐class logistic regression (OCLR) machine learning algorithm. All analyses in this section were performed using the SangerBox 3.0 platform [23].

### 2.7. Nomogram Construction

A multifactorial nomogram incorporating MYDGF expression and clinical characteristics was constructed using the “survival” and “rms” R packages. Calibration curves and decision curve analysis (DCA) were used to evaluate the predictive performance and clinical utility of the nomogram.

### 2.8. Prognostic Feature Selection Based on Machine Learning

Prognostic feature selection was performed using the glmnet R package. TCGA‐KIRC patients were randomly divided into a training cohort and an internal validation cohort at a 1:1 ratio. DEGs between the MYDGF‐H and MYDGF‐L groups were first subjected to univariate Cox regression analysis to identify genes significantly associated with patient survival. Survival time, survival status, and gene‐expression data were then integrated into a least absolute shrinkage and selection operator (LASSO) Cox regression model.

To reduce overfitting and determine the optimal penalty parameter, 10‐fold cross‐validation was performed. At the selected *λ* value, 19 MYDGF‐related prognostic genes were retained in the final model. A risk score was calculated for each patient as the weighted sum of the expression levels of these genes multiplied by their corresponding LASSO coefficients: risk score = *Σ* (coefficient_i × expression_i).

The complete list of the 19 genes, their LASSO coefficients, and the explicit risk‐score formula are provided in Table S1. Because comprehensive evaluation of the prognostic performance of this gene set, including risk‐score distribution, Kaplan–Meier analysis, time‐dependent ROC analysis, internal‐validation performance, and incremental value beyond MYDGF expression and standard clinicopathological variables, was not completed in the present study, this 19‐gene set is presented as a preliminary, hypothesis‐generating result requiring dedicated validation.

### 2.9. Cell Culture

Human renal cancer cell lines 769‐P and Caki‐1 and the normal human renal proximal tubular epithelial cell line HK‐2 were obtained from Procell (Wuhan, China). 769‐P cells were cultured in RPMI‐1640 medium, Caki‐1 cells in McCoy′s 5A medium, and HK‐2 cells in Minimum Essential Medium (MEM). All media were supplemented with 10% fetal bovine serum (FBS) and 1% penicillin–streptomycin. Cells were maintained at 37°C in a humidified atmosphere containing 5% CO_2_.

### 2.10. siRNA Transfection

MYDGF‐specific siRNAs (si‐MYDGF) and negative control siRNA (si‐NC) were synthesized by the Public Protein/Plasmid Library (PPL) (China). The sequences of MYDGF‐targeting siRNAs were as follows: siRNA#1, 5 ^′^‐GCGAAGACCACCAGCACUU‐3 ^′^; siRNA#2, 5 ^′^‐UCACACAGUUCAAGGCAGA‐3 ^′^; and siRNA#3, 5 ^′^‐AGGAAUUUGAAGUGACCAA‐3 ^′^. Cells were seeded in 6‐well plates and transfected at 50%–60% confluence using GP‐transfect‐Mate reagent. Follow‐up experiments were performed 48 h after transfection. Knockdown efficiency of all three siRNAs was first evaluated at the mRNA level; siRNA#2, which achieved the most efficient and reproducible knockdown and was further confirmed at the protein level by Western blotting, was selected for all subsequent functional experiments (proliferation, wound‐healing, and Transwell migration and invasion assays) and is hereafter referred to as si‐MYDGF.

### 2.11. Quantitative Real‐Time PCR

Total RNA was extracted using TRIGene Plus reagent, and cDNA was synthesized using StarScript Pro All‐in‐One RT Mix with gDNA Remover according to the manufacturer′s instructions. Quantitative real‐time PCR was performed using 2x RealStar Universal SYBR qPCR Mix. MYDGF primer sequences were as follows: forward, 5 ^′^‐GGCGTCGTGCATTCCTTCT‐3 ^′^; reverse, 5 ^′^‐CCATTGCTCATTGGTCCCTC‐3 ^′^. Relative gene expression was calculated using the 2^−*ΔΔ*Ct^ method.

### 2.12. Cell Proliferation Assay

Cell proliferation was assessed using the Cell Counting Kit‐8 (CCK‐8). Cells were seeded in 96‐well plates at a density of 2 × 10^3^ cells per well. At the indicated time points (0, 24, 48, and 72 h), CCK‐8 reagent was added and incubated for 2 h at 37°C. Absorbance at 450 nm was measured using a microplate reader. Growth curves were generated using GraphPad Prism 9.1.

### 2.13. Wound Healing Assay

Cells were cultured in 6‐well plates until reaching full confluence. Linear wounds were generated using a 200‐*μ*L pipette tip, and cell migration into the wound area was monitored and imaged using an optical microscope.

### 2.14. Transwell Migration and Invasion Assays

Cell migration and invasion were evaluated using Transwell chambers with 8‐*μ*m pore membranes. For migration assays, 2 × 10^4^ cells in serum‐free medium were seeded into the upper chamber, and medium containing 10% FBS was added to the lower chamber. After 48 h of incubation, migrated cells were fixed, stained with crystal violet, and counted in three randomly selected fields under 100x magnification. For invasion assays, the upper chamber was precoated with Matrigel for 3 h, and 5 × 10^4^ cells were seeded. Subsequent procedures were identical to those used for migration assays.

### 2.15. Western Blot Analysis

Western blotting was performed to assess MYDGF protein expression. Total protein was separated by SDS–PAGE and transferred onto PVDF membranes. Membranes were blocked with 5% nonfat milk and incubated overnight at 4°C with primary antibodies against MYDGF (Cat. No. 11353‐1‐AP, 1:1000) and *β*‐actin (Cat. No. 81115‐1‐RR, 1:10,000). After incubation with HRP‐conjugated secondary antibodies, signals were detected using an enhanced chemiluminescence kit and quantified using ImageJ software.

### 2.16. Statistical Analysis

All statistical analyses were performed in R (Version 4.1.0) and GraphPad Prism 9.1. Continuous variables between two groups were compared using the Wilcoxon rank‐sum (Mann–Whitney) test for nonnormally distributed data and the Student′s *t*‐test for normally distributed data, whereas comparisons among more than two groups used the Kruskal–Wallis test. Correlations between MYDGF expression and immune infiltration or functional signatures were assessed using Spearman′s correlation coefficient. Survival differences were evaluated by the log‐rank test, and Cox proportional hazards models were used for univariate and multivariate analyses. For analyses involving large numbers of simultaneous comparisons (e.g., immune‐infiltration and correlation analyses across multiple deconvolution algorithms, and immune checkpoint blockade response comparisons), *p* values were adjusted for multiple testing using the Benjamini–Hochberg false discovery rate or the Bonferroni method, as indicated in the corresponding figure legends. A two‐sided *p* < 0.05 was considered statistically significant; significance levels are denoted as  ^∗^ *p* < 0.05,  ^∗∗^ *p* < 0.01,  ^∗∗∗^ *p* < 0.001, and  ^∗∗∗∗^ *p* < 0.0001.

## 3. Results

### 3.1. Single‐Cell Analysis and Candidate Gene Screening

We analyzed the scRNA‐seq dataset GSE156632, which included tumor tissues from seven patients with ccRCC and five normal renal tissue samples. After stringent quality control to exclude cells with high mitochondrial gene content and low gene complexity, a total of 56,602 high‐quality cells were retained for downstream analyses.

Dimensionality reduction using t‐SNE and UMAP was performed to visualize the overall distribution of tumor and normal cells (Figure 1A). The Top 2000 highly variable genes were identified, and batch effects across samples were corrected using the Harmony algorithm. PCA was conducted, and clustering stability across resolutions was evaluated using Elbow plots and clustering tree visualization.

Figure 1Single‐cell transcriptomic landscape and identification of MYDGF‐related candidate genes in clear cell renal cell carcinoma. (A) Two‐dimensional t‐SNE and UMAP representations of 56,602 cells retained after quality control, showing the overall distribution of cells from ccRCC and adjacent normal renal tissues. (B) Unsupervised clustering of all cells visualized by t‐SNE and UMAP, yielding 42 distinct clusters at a resolution of 1.5. Colors indicate cluster assignment. (C) t‐SNE and UMAP visualization of the annotated single‐cell populations in ccRCC and normal renal tissues. Nine cell populations were identified, including epithelial cells, endothelial cells, fibroblast cells, macrophages, monocytes, dendritic cells, T cells, NK cells, and B cells. For downstream analyses, these populations were further grouped into six major lineages: epithelial/tumor, endothelial, myeloid, T/NK, B, and stromal cells. (D) Dot plot showing the expression of representative marker genes across the initial unsupervised clusters, used as references during annotation before consolidation into the six final cell types. (E) Dot plot of canonical marker gene expression across the six final annotated cell types, including epithelial/tumor, immune, and stromal populations. Dot size reflects the proportion of expressing cells, and color intensity denotes average expression levels. (F) Venn diagram illustrating the overlap of genes derived from malignant epithelial cells through copy‐number variation analysis, differential expression analysis, and survival association, resulting in 30 candidate genes selected for subsequent analyses.

**Figure:**
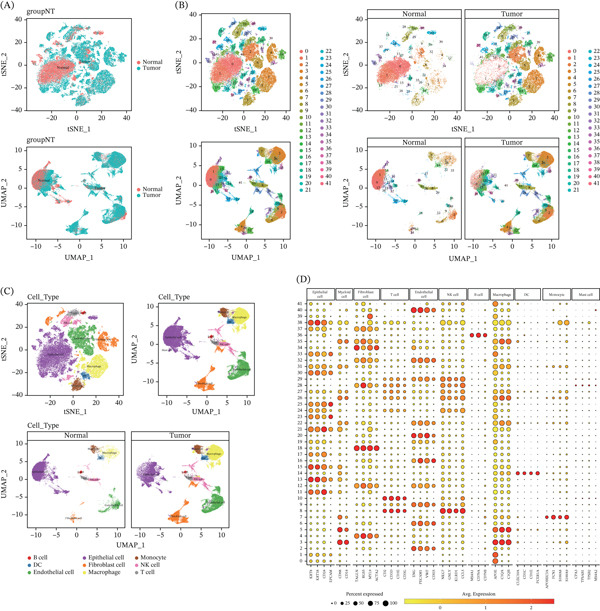
(A)

**Figure:**
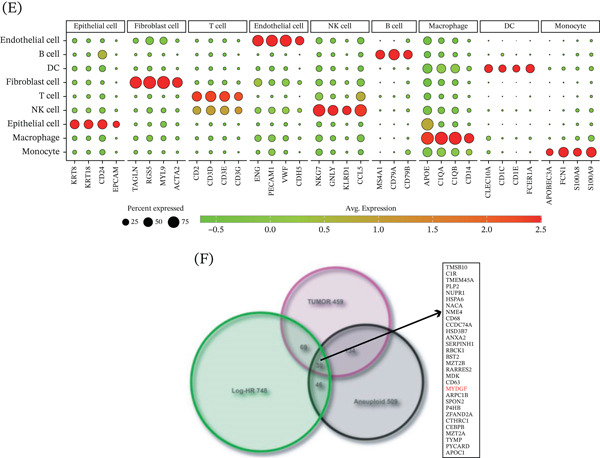
(B)

Based on these analyses, a clustering resolution of 1.5 was selected, yielding 42 distinct cell clusters. These clusters were visualized using t‐SNE and UMAP, demonstrating clear separation and sample distribution patterns across tumor and normal tissues (Figure 1B).

Cell‐type annotation was performed based on established canonical marker genes. Nine cell populations were initially identified, including epithelial cells, endothelial cells, fibroblast cells, macrophages, monocytes, dendritic cells, T cells, NK cells, and B cells (Figure 1C–E). For downstream analyses and consistent presentation, these populations were further summarized into six major lineages: epithelial/tumor cells, endothelial cells, myeloid cells, T/NK cells, B cells, and stromal cells.

To further characterize malignant epithelial populations, DEGs between epithelial cells from tumor and normal tissues were identified (log2FC > 0.585, FDR < 0.05). CNV analysis using the copyKAT algorithm revealed elevated aneuploidy levels in tumor epithelial cells compared with diploid normal epithelial cells. Integration with TCGA transcriptomic data further refined this gene set, resulting in 30 candidate genes that were significantly upregulated in tumors and negatively associated with patient prognosis (Figure 1F).

### 3.2. MYDGF Expression Landscape and Clinical Significance in ccRCC

**Figure 2:**
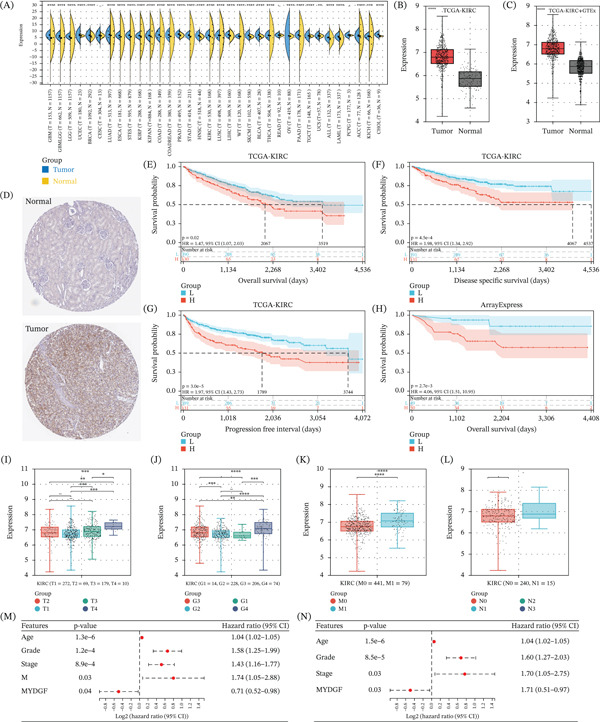
MYDGF expression, prognostic value, and clinical relevance in clear cell renal cell carcinoma. (A) Pan‐cancer overview of MYDGF mRNA expression across different tumor types based on TCGA data. (B) MYDGF expression levels in ccRCC tumor tissues and normal renal tissues from the TCGA‐KIRC cohort. (C) MYDGF expression in ccRCC tumors and normal renal tissues using combined TCGA and GTEx datasets. (D) Representative immunohistochemical staining of MYDGF in normal kidney tissue and ccRCC tissue obtained from the Human Protein Atlas (HPA). (E–G) Kaplan–Meier survival analyses illustrating the association between MYDGF expression and overall survival (OS), disease‐specific survival (DSS), and progression‐free interval (PFI) in patients from the TCGA‐KIRC cohort. (H) Kaplan–Meier survival curves validating the prognostic relevance of MYDGF expression in an independent ccRCC cohort from ArrayExpress. (I–L) Relationship between MYDGF expression and clinicopathological characteristics of ccRCC, including tumor stage, histological grade, and metastatic status. (M, N) Forest plots from univariate and multivariate Cox proportional hazards regression analyses assessing the prognostic impact of MYDGF expression alongside clinical variables in ccRCC. Statistical analyses were performed as described in the Methods section. Box plots indicate median values with interquartile ranges. *p* < 0.05, *p* < 0.01, *p* < 0.001, *p* < 0.0001; ns, not significant.

Pan‐cancer analyses showed that MYDGF displayed heterogeneous expression patterns across tumor types (Figure 2A). In ccRCC, MYDGF expression was significantly higher in tumor tissues than in normal renal tissues in TCGA‐KIRC (Figure 2B), and this finding was supported by the combined TCGA/GTEx comparison (Figure 2C). Immunohistochemistry data from the HPA further suggested elevated MYDGF protein staining in renal cancer tissues (Figure 2D).

Using the optimal cutoff, patients were stratified into MYDGF‐H and MYDGF‐L groups. Kaplan–Meier analyses demonstrated that higher MYDGF expression was associated with worse outcomes (OS, disease‐specific survival [DSS], and progression‐free interval [PFI]) in TCGA‐KIRC, and this prognostic association was validated in an independent cohort (Figure 2E–H). MYDGF expression also increased with more advanced clinicopathological features, including higher T stage and pathological grade, and was associated with metastatic status (Figure 2I–L).

To further clarify the genomic context of MYDGF expression, we assessed its relationship with several molecular indices. MYDGF expression correlated positively with TMB and inversely with tumor purity (Figure S1A,C), whereas no meaningful associations were observed with MSI or mutant‐allele tumor heterogeneity (MATH) scores (Figure S1B,D). Comparison of mutation landscapes between MYDGF‐H and MYDGF‐L tumors revealed distinct differences in several frequently altered genes, most notably PBRM1, SETD2, BAP1, TTN, and PRKDC (Figure S1E).

Cox regression analyses further indicated that MYDGF retained prognostic significance after adjusting for clinical covariates, supporting its role as an independent predictor in ccRCC (Figure 2M,N).

### 3.3. Functional Features of MYDGF‐Associated DEGs

**Figure 3:**
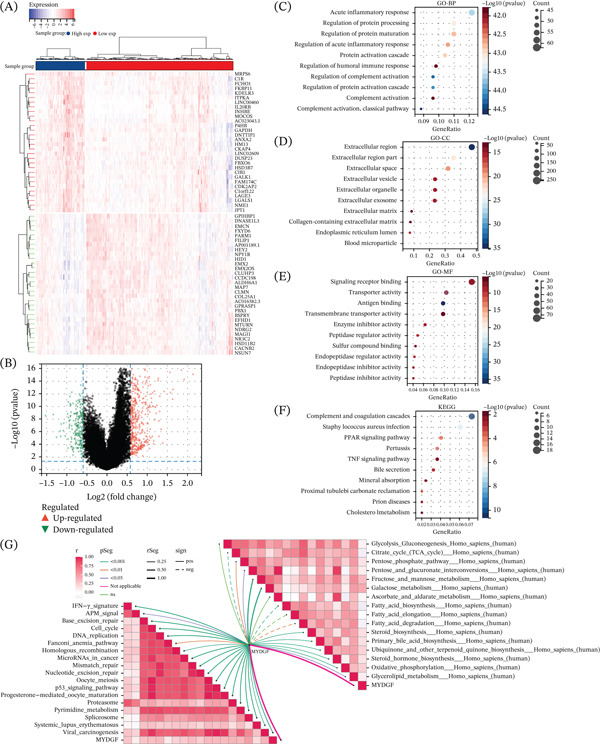
Functional enrichment and pathway characteristics of MYDGF‐associated differentially expressed genes in ccRCC. (A) Heatmap showing the expression patterns of the Top 30 differentially expressed genes (DEGs) between the MYDGF high‐ and low‐expression groups in ccRCC samples. (B) Volcano plot illustrating the global distribution of DEGs between the MYDGF high‐ and low‐expression groups, with upregulated and downregulated genes highlighted. (C) Gene Ontology (GO) biological process (BP) enrichment analysis of MYDGF‐associated DEGs. (D) GO cellular component (CC) enrichment analysis of MYDGF‐associated DEGs. (E) GO molecular function (MF) enrichment analysis of MYDGF‐associated DEGs. (F) Kyoto Encyclopedia of Genes and Genomes (KEGG) pathway enrichment analysis of MYDGF‐associated DEGs. (G) Correlation network depicting the associations between MYDGF expression and immune‐related as well as metabolic‐related functional terms, based on enrichment and correlation analyses.

Differential expression analysis between MYDGF‐H and MYDGF‐L groups identified 464 upregulated and 207 downregulated genes. The expression patterns of the top DEGs were visualized by heatmap, and the overall DEG distribution was shown by volcano plot (Figure 3A,B).

GO enrichment analyses indicated that MYDGF‐associated DEGs were enriched in biological processes related to inflammatory and immune regulation (Figure 3C), with cellular component terms predominantly mapped to extracellular compartments (Figure 3D). Molecular function terms were mainly enriched in receptor binding and immune‐related activities (Figure 3E). KEGG analysis highlighted complement and coagulation cascades and the PPAR signaling pathway among the top enriched pathways (Figure 3F). In addition, correlation network analyses suggested positive associations between MYDGF expression and multiple immune‐related signatures (e.g., IFN‐*γ* signature and antigen processing/presentation machinery), whereas negative associations were observed with several metabolic terms (Figure 3G).

### 3.4. Association of MYDGF With Immune Infiltration and Immune Checkpoint–Related Genes

**Figure 4:**
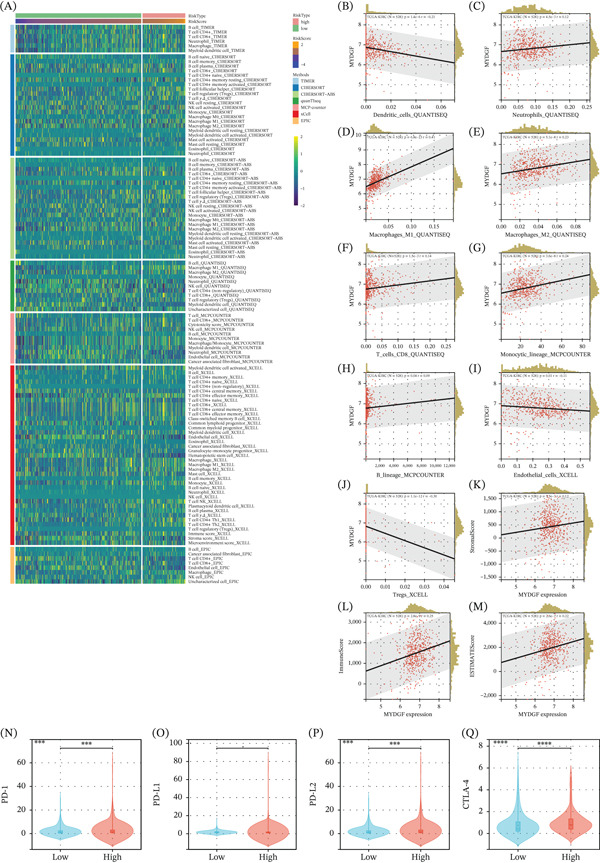
Association between MYDGF expression and the immune microenvironment in clear cell renal cell carcinoma. (A) Heatmap illustrating immune cell infiltration patterns in ccRCC samples stratified by high (MYDGF‐H) and low (MYDGF‐L) MYDGF expression. Immune cell abundances were estimated using multiple computational algorithms, as indicated. (B–M) Scatter plots showing the correlations between MYDGF expression and the inferred infiltration levels of neutrophils, M1 macrophages, M2 macrophages, CD8^+^ T cells, monocytes, B cells, dendritic cells, endothelial cells, and regulatory T cells across ccRCC samples. Shaded areas represent confidence intervals for the fitted regression lines. (N–Q) Violin plots comparing the expression levels of immune checkpoint–related genes, including PDCD1 (PD‐1), CD274 (PD‐L1), PDCD1LG2 (PD‐L2), and CTLA‐4, between MYDGF‐H and MYDGF‐L groups. Statistical analyses were performed as described in the Methods section.

To characterize the immune microenvironment of ccRCC, immune cell infiltration was estimated using multiple computational algorithms, including TIMER, CIBERSORT, CIBERSORT‐ABS, quanTIseq, MCP‐counter, xCell, and EPIC [7]. Across these independent methods, MYDGF expression showed consistent associations with immune infiltration patterns. Specifically, higher MYDGF expression was positively correlated with inferred infiltration levels of neutrophils, macrophages (M1 and M2), CD8^+^ T cells, monocytes, and B cells, whereas negative correlations were observed with dendritic cells as well as stromal‐related components such as endothelial cells and regulatory T cells (Figure 4A–J).

In addition to individual immune cell populations, MYDGF expression was positively associated with global immune‐related indices, including immune score, stromal score, and ESTIMATE score (Figure 4K–M).

Analysis of immune checkpoint–related genes revealed that PDCD1 (PD‐1), PDCD1LG2 (PD‐L2), and CTLA‐4 were significantly upregulated in the MYDGF high‐expression group, whereas CD274 (PD‐L1) did not show a clear difference between MYDGF subgroups (Figure 4N–Q). Together, these results suggest that MYDGF expression is associated with the immune contexture and immune checkpoint–related features of ccRCC.

### 3.5. Immunotherapy‐Related Characteristics, Drug Sensitivity, and Prognostic Modeling

**Figure 5:**
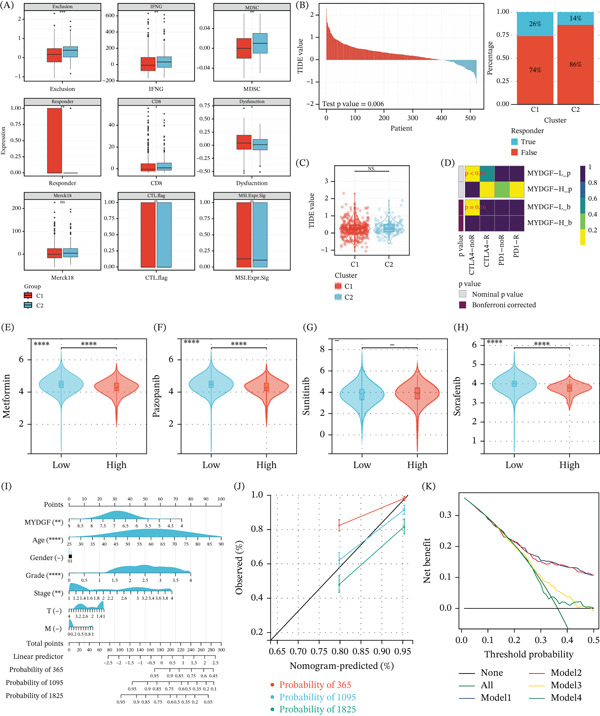
Immunotherapy‐related characteristics, drug sensitivity, and prognostic modeling associated with MYDGF expression in clear cell renal cell carcinoma. (A) Box plots comparing tumor immune–related indicators between MYDGF low‐expression (MYDGF‐L, C1) and high‐expression (MYDGF‐H, C2) groups in ccRCC. (B) Left panel shows the distribution of Tumor Immune Dysfunction and Exclusion (TIDE) scores in MYDGF‐L and MYDGF‐H groups, whereas the right panel summarizes the predicted proportions of immunotherapy responders and nonresponders in each group based on the TIDE model. (C) Violin plot illustrating the difference in TIDE scores between MYDGF‐L (C1) and MYDGF‐H (C2) groups. (D) Heatmaps displaying the nominal *p* values and Bonferroni‐corrected *p* values derived from comparisons of predicted responses to immune checkpoint blockade targeting PD‐1 and CTLA‐4 between MYDGF‐H and MYDGF‐L groups. (E–H) Violin plots comparing the predicted sensitivities to representative targeted therapeutic agents between MYDGF‐H and MYDGF‐L groups. (I) Nomogram integrating MYDGF expression and clinicopathological variables to estimate the survival probability of patients with ccRCC. (J) Calibration curves evaluating the agreement between nomogram‐predicted survival probabilities and observed outcomes. (K) Decision curve analysis (DCA) assessing the potential clinical utility of the MYDGF‐based nomogram in comparison with alternative predictive models. Statistical analyses were performed as described in the Methods section.

TIDE‐based analyses were performed to evaluate immunotherapy‐related features under MYDGF‐based stratification. MYDGF expression was associated with multiple immunotherapy‐relevant indicators (Figure 5A), and the predicted proportion of immunotherapy responders was lower in the MYDGF‐H group than in the MYDGF‐L group (Figure 5B). Notably, the overall TIDE score itself did not differ markedly between groups (Figure 5C). Comparisons of predicted responses to PD‐1 and CTLA‐4 blockade (with nominal and Bonferroni‐corrected *p* values) are summarized in Figure 5D. It should be emphasized that these TIDE‐based results are computational predictions and are therefore exploratory in nature; they do not constitute direct evidence of clinical response and require validation in independent ccRCC cohorts treated with immune checkpoint blockade.

We further evaluated predicted drug sensitivity and observed group differences for several agents, including metformin, sorafenib, and pazopanib (Figure 5E–H). A nomogram integrating MYDGF expression with clinicopathological variables was constructed (Figure 5I), and calibration curves indicated good agreement between predicted and observed survival probabilities (Figure 5J). DCA suggested that the integrated model provided improved clinical utility compared with alternative models (Figure 5K).

### 3.6. Experimental Validation of MYDGF Function and Construction of a MYDGF‐Derived Prognostic Signature

**Figure 6:**
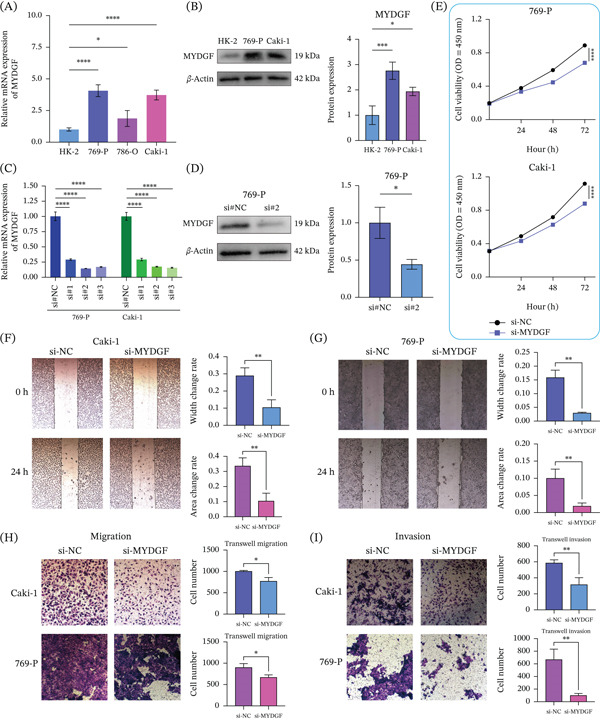
Functional validation of MYDGF in clear cell renal cell carcinoma cells. (A) Quantitative real‐time PCR (qRT‐PCR) analysis of MYDGF mRNA expression in ccRCC cell lines (769‐P and Caki‐1) compared with the normal renal tubular epithelial cell line HK‐2. (B) Western blot analysis showing MYDGF protein expression in ccRCC cell lines and HK‐2 cells. Representative blots and corresponding quantitative analyses are shown. (C, D) qRT‐PCR and Western blot analyses confirming the knockdown efficiency of MYDGF following siRNA transfection in 769‐P and Caki‐1 cells. (E) Cell proliferation assessed by CCK‐8 assays in 769‐P and Caki‐1 cells transfected with control siRNA (si‐NC) or MYDGF‐targeting siRNA (si‐MYDGF). (F, G) Wound healing assays evaluating the migratory capacity of 769‐P and Caki‐1 cells after MYDGF knockdown at the indicated time points. (H, I) Transwell migration and invasion assays assessing the effects of MYDGF knockdown on the migratory and invasive abilities of 769‐P and Caki‐1 cells. All experiments were performed independently at least three times. Quantitative data are presented as mean ± standard deviation (SD). Statistical significance was determined using appropriate tests. *p* < 0.05, *p* < 0.01, *p* < 0.001, *p* < 0.0001.

To experimentally validate the functional role of MYDGF, its expression was first assessed in ccRCC cell lines. qRT‐PCR confirmed higher MYDGF mRNA levels in ccRCC cell lines compared with the normal renal tubular epithelial cell line HK‐2 (Figure 6A). Western blotting further verified elevated MYDGF protein expression in representative ccRCC cell lines (Figure 6B).

After siRNA‐mediated knockdown of MYDGF in 769‐P and Caki‐1 cells, both qRT‐PCR and Western blot analyses confirmed efficient downregulation (Figure 6C,D). Functionally, MYDGF knockdown significantly inhibited cell proliferation as assessed by CCK‐8 assays (Figure 6E). In addition, wound healing and Transwell migration assays consistently showed reduced migratory capacity after MYDGF silencing (Figure 6F–H), and Transwell invasion assays demonstrated markedly decreased invasive ability (Figure 6I). These findings support a protumorigenic role of MYDGF in promoting proliferative and invasive behaviors in ccRCC cells.

To explore its prognostic implications, we screened MYDGF‐associated DEGs using univariate Cox regression and identified multiple genes linked to patient survival (Figure S2A). LASSO Cox analysis was then applied to refine the gene set, with cross‐validation determining the optimal *λ* value (Figure S2B,C). This process yielded a preliminary 19‐gene MYDGF‐related gene set; the complete list of genes and their corresponding LASSO coefficients, together with the risk‐score formula, is provided in Table S1. Because a comprehensive performance evaluation of this gene set—including the risk‐score distribution, Kaplan–Meier survival analysis, time‐dependent ROC analysis, and assessment in the internal validation cohort, as well as a comparison with MYDGF expression and standard clinicopathological variables—has not yet been completed, we present it as a preliminary, hypothesis‐generating result rather than a finalized prognostic model, and it will require dedicated validation in future studies.

## 4. Discussion

MYDGF was initially identified as a stress‐responsive secreted factor with protective roles in nonneoplastic conditions. Previous studies have demonstrated that MYDGF contributes to cardiac repair after myocardial infarction by limiting cardiomyocyte apoptosis and promotes metabolic homeostasis by improving glucose tolerance and lipid metabolism, partly through regulation of enteroendocrine signaling [8, 24, 25]. These findings indicate that MYDGF exerts pleiotropic biological effects beyond cardiovascular and metabolic systems, raising the possibility that it may also participate in pathological processes such as tumor development and progression [8]. Notably, a recent study demonstrated that tubular MYDGF slows the progression of chronic kidney disease by maintaining mitochondrial homeostasis, highlighting its protective role in nonneoplastic renal conditions [26], which stands in contrast to its protumorigenic role observed in our study.

Myeloid‐derived cells and their secreted factors have become a growing focus in studies of the TME. Interactions between myeloid populations and tumor cells are now recognized as key determinants of immune suppression, angiogenesis, and metastatic potential [2, 6]. Cytokines within the IL‐27 family, including IL‐30, have been reported to modulate tumor progression by influencing myeloid cell function and immune cell balance in several malignancies [27–29]. Although MYDGF is distinct from classical cytokines, its myeloid‐derived origin suggests that it may participate in related regulatory processes within the TME. However, the role of MYDGF in cancer biology has remained largely unexplored, particularly in renal malignancies [8].

ccRCC represents a clinically challenging tumor entity characterized by marked intratumoral heterogeneity and variable responses to targeted therapy and immune checkpoint blockade [1–3]. Despite advances in VEGF‐targeted agents and immunotherapy, a substantial proportion of patients experience primary resistance or disease progression during treatment [3, 30]. These limitations highlight the need to better understand how tumor‐intrinsic factors interact with the microenvironment to influence therapeutic responsiveness [2, 3, 6]. In the present study, MYDGF was identified through a systematic screening strategy as a candidate risk gene with elevated expression in ccRCC and consistent association with adverse clinical outcomes. By integrating single‐cell transcriptomics, bulk multiomics analyses, and in vitro functional assays, we provide a comprehensive characterization of MYDGF expression patterns, biological relevance, and clinical significance in ccRCC.

Methodologically, this work illustrates the value of integrating single‐cell resolution with machine learning–based modeling in biomarker discovery. Single‐cell data enabled precise localization of MYDGF‐expressing malignant subpopulations, whereas computational modeling identified downstream prognostic signatures that could not be resolved using bulk datasets alone. This integrated framework may be broadly applicable to the identification of immune‐diagnostic markers and therapeutic targets across other solid tumors [12, 31, 32].

Using scRNA‐seq data, we observed that MYDGF expression was associated with differences in the composition of the tumor immune microenvironment. Tumors with high MYDGF expression exhibited increased infiltration of neutrophils, macrophages, monocytes, and CD8^+^ T cells, accompanied by reduced dendritic cell and regulatory T‐cell signatures. Neutrophils and macrophages, particularly M2‐polarized subsets, are well‐recognized contributors to immune suppression in ccRCC through the secretion of inhibitory cytokines and modulation of antigen presentation [6, 33]. As a myeloid‐derived factor, we speculate that MYDGF might facilitate the recruitment and functional polarization of myeloid‐derived suppressor cells (MDSCs), which are known to exert potent protumorigenic and immunosuppressive effects in the ccRCC TME [34, 35]. Although CD8^+^ T cells are central mediators of antitumor immunity, accumulating evidence suggests that, in ccRCC, these cells often display functional exhaustion rather than effective cytotoxic activity, especially in myeloid‐rich microenvironments. Consistent with this interpretation, MYDGF‐H tumors showed elevated expression of immune checkpoint molecules, including PDCD1 (PD‐1), PDCD1LG2 (PD‐L2), and CTLA‐4, indicating that MYDGF‐associated immune infiltration may reflect an immune‐infiltrated but functionally restrained tumor state [7].

Immune checkpoint expression and immune cell abundance did not translate into favorable immunotherapy response predictions in MYDGF‐H tumors. TIDE‐based analyses suggested that patients with elevated MYDGF expression were less likely to benefit from immune checkpoint blockade, despite higher immune and ESTIMATE scores [22]. This apparent discordance has been increasingly reported in ccRCC and is thought to arise from signaling‐driven immune dysfunction, in which immune cells are present but ineffective [7]. As Braun et al. [7] elucidated through single‐cell analysis, this progressive immune dysfunction is a hallmark of advanced ccRCC, where the TME transitions toward a more terminal, exhausted state despite high immune infiltration. Within this context, MYDGF may serve as a marker of a microenvironmental state characterized by immune infiltration coupled with impaired antitumor activity, thereby contributing to adaptive resistance to immunotherapy [3, 30].

At the signaling level, MYDGF‐associated gene expression patterns were consistently enriched in pathways related to PI3K/AKT and MAPK signaling. These pathways are central regulators of cell survival, metabolic adaptation, angiogenesis, and therapeutic resistance in ccRCC [36, 37]. Although direct pathway activation was not experimentally assessed in this study, the observed enrichment patterns, together with prior reports of MYDGF‐mediated AKT activation in nontumor settings, suggest that MYDGF may be linked to a signaling context that facilitates tumor cell tolerance to therapeutic stress [26]. Rather than acting as a classical oncogenic driver, MYDGF may therefore reflect a permissive signaling environment that supports tumor persistence under treatment pressure [3].

MYDGF expression was also linked to differential predicted sensitivity to several targeted agents commonly used in ccRCC, including sorafenib and pazopanib. These agents exert their antitumor effects, at least in part, through modulation of angiogenic and survival‐related signaling pathways [38]. The observed associations do not imply direct drug targeting by MYDGF, but instead support the idea that MYDGF expression may indicate broader signaling states that influence treatment responsiveness [3]. The observed negative association between MYDGF and metabolic pathways aligns with the established view of ccRCC as a metabolic disease characterized by redirected nutrient flux [39, 40]. As a speculative hypothesis that remains to be tested, MYDGF might act as a metabolic‐signaling rheostat that shifts the cellular program from oxidative metabolism toward signaling‐driven survival under stress. This observation aligns with findings in hepatocellular carcinoma, where hypoxia‐induced MYDGF has been shown to promote sorafenib resistance by driving TME remodeling and specific signaling reprogramming [9].

In addition to its associations with immune and signaling features, MYDGF expression correlated with adverse clinicopathological characteristics, genomic alterations, and poor survival outcomes. High MYDGF expression was associated with advanced tumor stage, higher grade, increased TMB, and altered mutation frequencies of key ccRCC genes such as PBRM1 and SETD2 [41, 42]. Specifically, recent evidence indicates that PBRM1 loss or alternative splicing can mediate resistance to PD‐1 blockade [41], whereas SETD2 deficiency promotes renal cancer development via metabolic reprogramming [42], further supporting the clinical relevance of the MYDGF‐associated genomic landscape. Functional experiments further demonstrated that MYDGF contributes to malignant phenotypes at the cellular level, as silencing MYDGF significantly suppressed ccRCC cell proliferation, migration, and invasion. Given that MYDGF‐associated DEGs were enriched in extracellular exosomes, we speculate, as a hypothesis that was not directly tested in the present study, that MYDGF might be conveyed by exosomal vesicles to participate in intercellular communication; whether this contributes to TME remodeling or premetastatic niche formation remains entirely unverified and will require dedicated experimental validation [43]. These observations support a role for MYDGF in ccRCC progression through coordinated effects on tumor cell behavior and the surrounding microenvironment.

Overall, our data position MYDGF as a clinically relevant factor that is associated with tumor‐microenvironment composition and signaling features that may relate to adaptive therapeutic resistance in ccRCC. Rather than serving solely as a prognostic biomarker, MYDGF appears to reflect a coordinated immune and signaling context that favors tumor persistence during therapy. This framework provides a rationale for future mechanistic studies aimed at dissecting MYDGF‐associated signaling networks and evaluating whether combinatorial strategies targeting these pathways, together with immune or targeted therapies, may improve outcomes in selected ccRCC patient subsets [3, 30].

Several limitations should be acknowledged. The mechanistic links between MYDGF and specific signaling pathways were inferred from enrichment analyses rather than direct functional validation, and in vivo studies were not performed. Future investigations incorporating pathway‐level experiments and animal models will be required to fully elucidate the role of MYDGF in ccRCC progression and therapeutic resistance.

## 5. Conclusion

In summary, by integrating single‐cell profiling, bulk multiomics analyses, and in vitro experiments, we identify MYDGF as a malignant epithelium–associated factor that is associated with an immune‐infiltrated yet functionally dysregulated TME in ccRCC. Its expression aligns with adverse clinicopathological features, key genomic alterations, and signaling programs linked to therapeutic tolerance, whereas MYDGF knockdown suppresses proliferative and invasive phenotypes in vitro. These findings highlight MYDGF as a biologically and clinically relevant marker associated with the immune and signaling microenvironment in ccRCC and provide a foundation for future mechanistic and translational studies.

**Nomenclature**
APM: antigen processing and presentation machinery
BP: biological process
CC: cellular component
CCK‐8: Cell Counting Kit‐8
ccRCC: clear cell renal cell carcinoma
CTLA‐4: cytotoxic T‐lymphocyte‐associated protein 4
DCA: decision curve analysis
DEG: differentially expressed gene
DSS: disease‐specific survival
GEO: Gene Expression Omnibus
GLP‐1: glucagon‐like peptide‐1
GO: Gene Ontology
GTEx: Genotype‐Tissue Expression
HPA: Human Protein Atlas
HR: hazard ratio
IFN‐*γ*
: interferon gamma
KEGG: Kyoto Encyclopedia of Genes and Genomes
KNN: k‐nearest neighbors
LASSO: least absolute shrinkage and selection operator
MATH: mutant‐allele tumor heterogeneity
MF: molecular function
mRNAsi: mRNA expression‐based stemness index
MSI: microsatellite instability
MYDGF: myeloid‐derived growth factor
OCLR: one‐class logistic regression
OS: overall survival
PCA: principal component analysis
PD‐1: programmed cell death protein 1
PD‐L1: programmed death‐ligand 1
PD‐L2: programmed death‐ligand 2
PFI: progression‐free interval
PVDF: polyvinylidene difluoride
QC: quality control
qRT‐PCR: quantitative real‐time reverse transcription polymerase chain reaction
RCC: renal cell carcinoma
scRNA‐seq: single‐cell RNA sequencing
si‐MYDGF: siRNA targeting MYDGF
si‐NC: small interfering negative control
siRNA: small interfering RNA
t‐SNE: t‐distributed Stochastic Neighbor Embedding
TCGA: The Cancer Genome Atlas
TCGA‐KIRC: The Cancer Genome Atlas Kidney Renal Clear Cell Carcinoma
TIDE: Tumor Immune Dysfunction and Exclusion
TMB: tumor mutational burden
TME: tumor microenvironment
UMAP: Uniform Manifold Approximation and Projection
WB: Western blotting

## Author Contributions

Y.X.: conceptualization, methodology, software, formal analysis, visualization, and writing–original draft. G.L.: methodology, software, validation, investigation, data curation, and writing–original draft. Y.X. and G.L. contributed equally to this work. X.R.: data curation, investigation, resources, and writing–review and editing. X.Q.: validation, resources, supervision, and writing–review and editing. G.W.: conceptualization, supervision, project administration, funding acquisition, and writing–review and editing.

## Funding

This study was supported by the Dalian Life and Health Field Guidance Plan (2024ZDJH01PT068), Open Fund Project of the Provincial Key Laboratory of Multidimensional Omics and Molecular Enzymology (2025YB007), and Scientific Research Project from the Educational Department of Liaoning Province (LJ212410161044).

## Disclosure

All authors have reviewed and approved the final version of the manuscript.

## Ethics Statement

This study did not involve any experiments on humans or animals. Ethical approval and informed consent were therefore not required.

## Consent

The authors have nothing to report.

## Conflicts of Interest

The authors declare no conflicts of interest.

## Supporting Information

Additional supporting information can be found online in the Supporting Information section.

## Supporting information


**Supporting Information 1** Figure S1: Association between MYDGF expression and genomic characteristics in clear cell renal cell carcinoma. (A–D) Scatter plots showing the correlations between MYDGF expression and key genomic features in ccRCC, including tumor mutational burden (TMB), microsatellite instability (MSI), tumor purity, and intratumoral heterogeneity, as indicated. Solid lines represent fitted regression trends, and shaded areas denote confidence intervals. (E) Oncoplot depicting the mutation landscape of the Top 15 genes with the highest mutation frequencies in ccRCC patients stratified by MYDGF high (MYDGF‐H) and low (MYDGF‐L) expression groups. Different colors indicate distinct mutation types, and the bar plots summarize mutation frequencies across samples.

**Supporting Information 2** Figure S2: Construction of a MYDGF‐related prognostic gene signature in clear cell renal cell carcinoma. (A) Forest plot showing the hazard ratios (HRs) and 95% confidence intervals (CIs) of the Top 50 MYDGF‐associated genes significantly correlated with patient prognosis based on univariate Cox proportional hazards regression analysis. (B) Least absolute shrinkage and selection operator (LASSO) Cox regression coefficient profiles of candidate MYDGF‐associated genes, illustrating the trajectories of regression coefficients with varying penalty parameters (*λ*). (C) Cross‐validation plot for LASSO Cox regression showing the partial likelihood deviance as a function of log (*λ*). The dashed vertical line indicates the optimal value of *λ* selected by minimum criteria.

**Supporting Information 3** Table S1: The 19 MYDGF‐related genes retained by LASSO Cox regression and their corresponding regression coefficients.

## Data Availability

The data that support the findings of this study are available from the corresponding authors upon reasonable request.
