# Supplementary figures and images for: Single‐Cell and Machine Learning Analyses Identify MYDGF as an Immune‐Related Biomarker Associated With the Tumor Microenvironment in Clear Cell Renal Cell Carcinoma
Source: Hum Mutat. 2026 Jul 25;2026:5262666. doi: 10.1155/humu/5262666 (PMC13401162; doi:10.1155/humu/5262666)

A

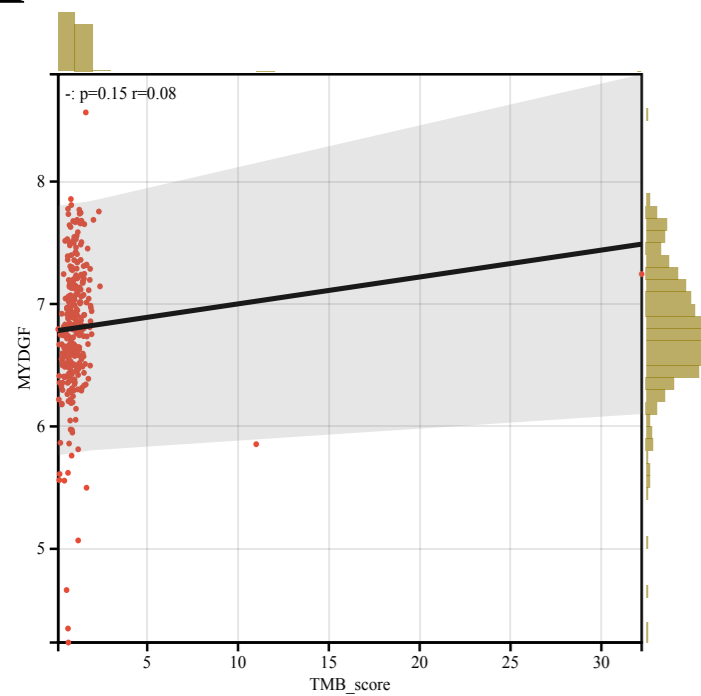

B

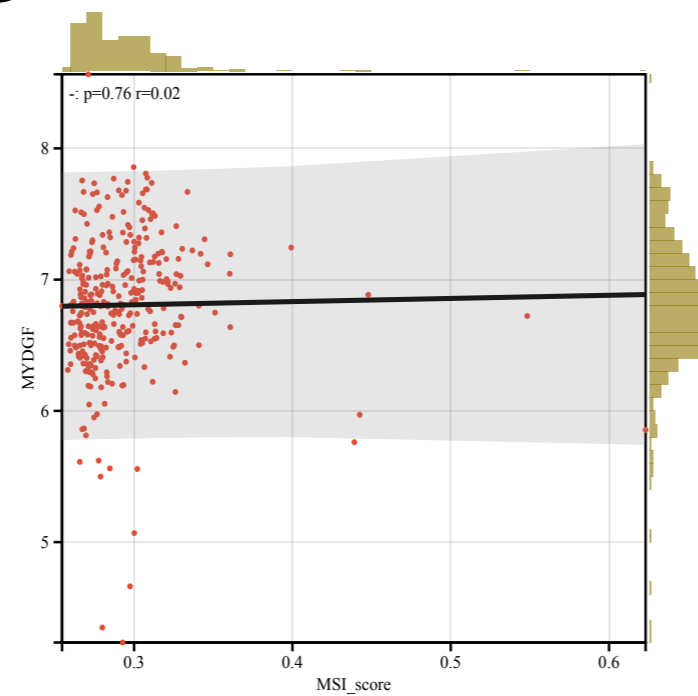

C

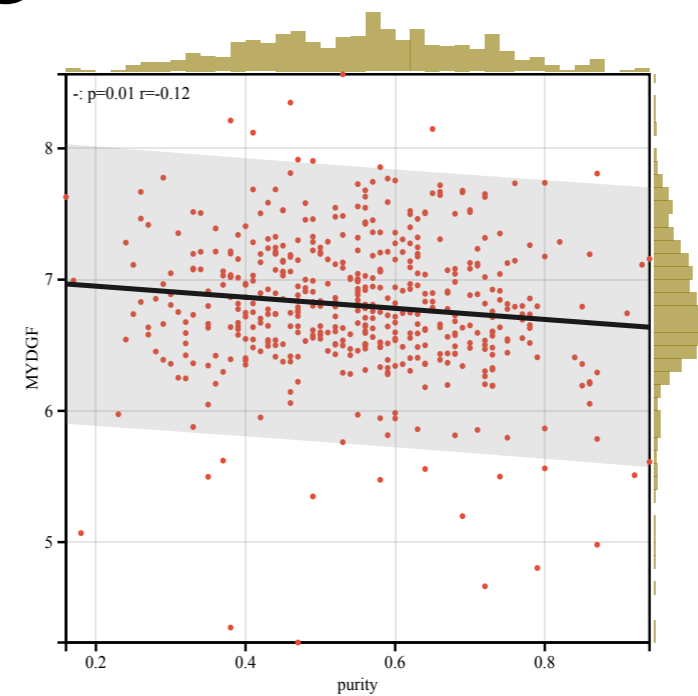

D

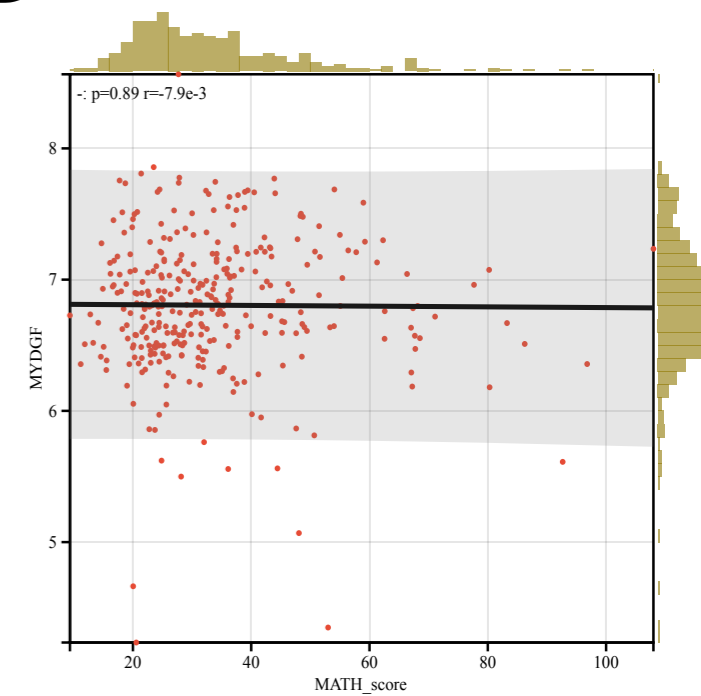

E

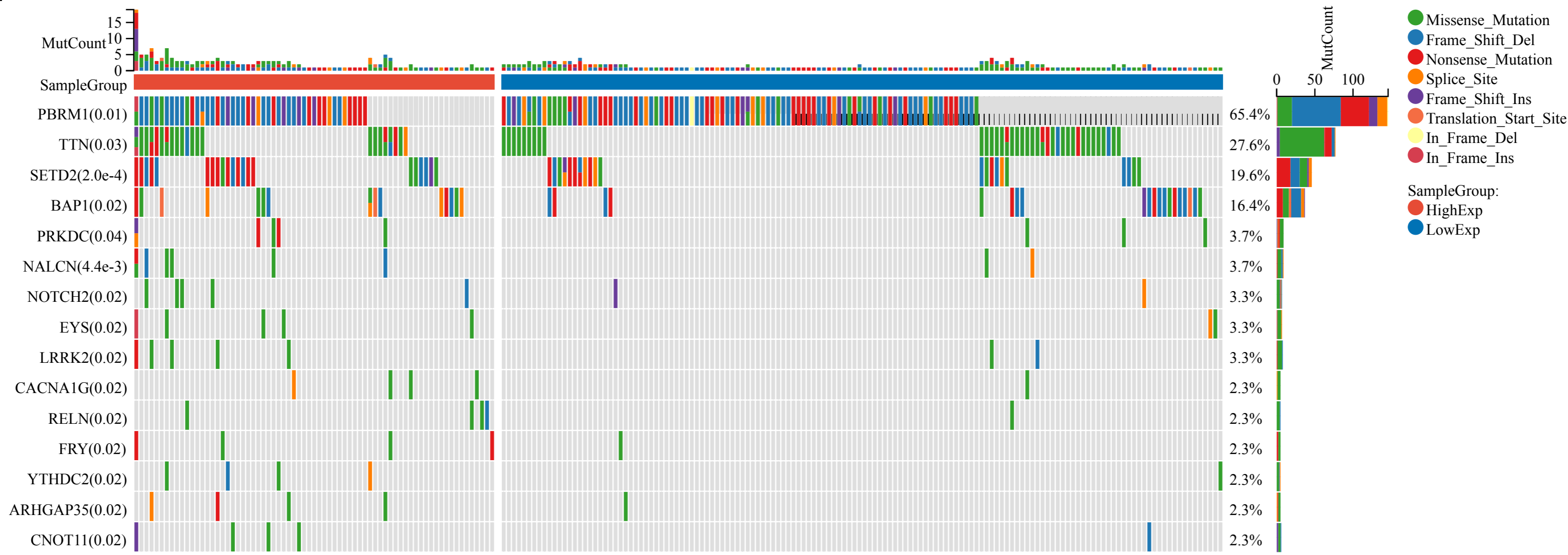

Supplement: Supplementary file 1 — Supporting Information 1 Figure S1: Association between MYDGF expression and genomic characteristics in clear cell renal cell carcinoma. (A–D) Scatter plots showing the correlations between MYDGF expression and key genomic features in ccRCC, including tumor mutational burden (TMB), microsatellite instability (MSI), tumor purity, and intratumoral heterogeneity, as indicated. Solid lines represent fitted regression trends, and shaded areas denote confidence intervals. (E) Oncoplot depicting the mutation landscape of the Top 15 genes with the highest mutation frequencies in ccRCC patients stratified by MYDGF high (MYDGF‐H) and low (MYDGF‐L) expression groups. Different colors indicate distinct mutation types, and the bar plots summarize mutation frequencies across samples. [file HUMU-2026-5262666-s001.pdf]

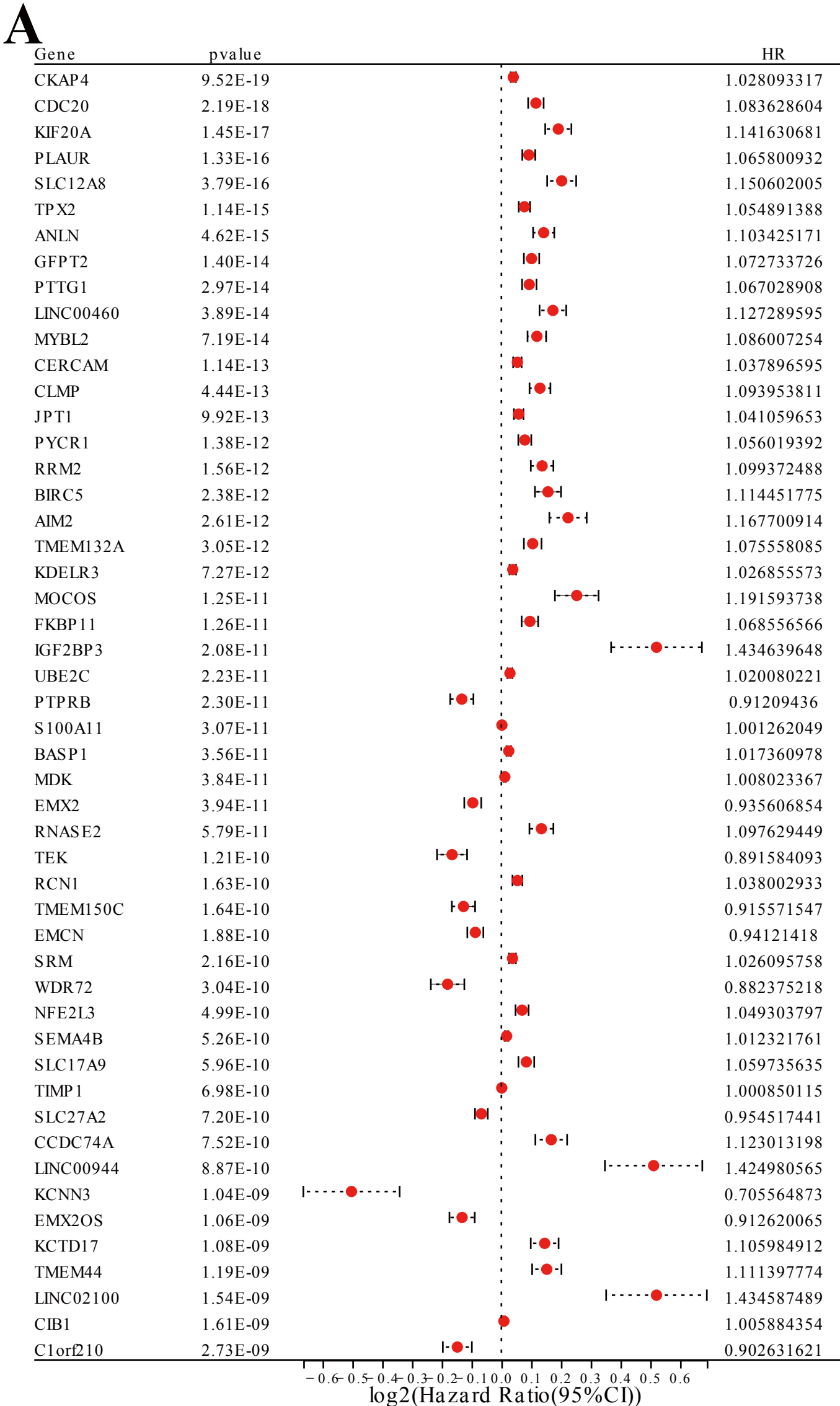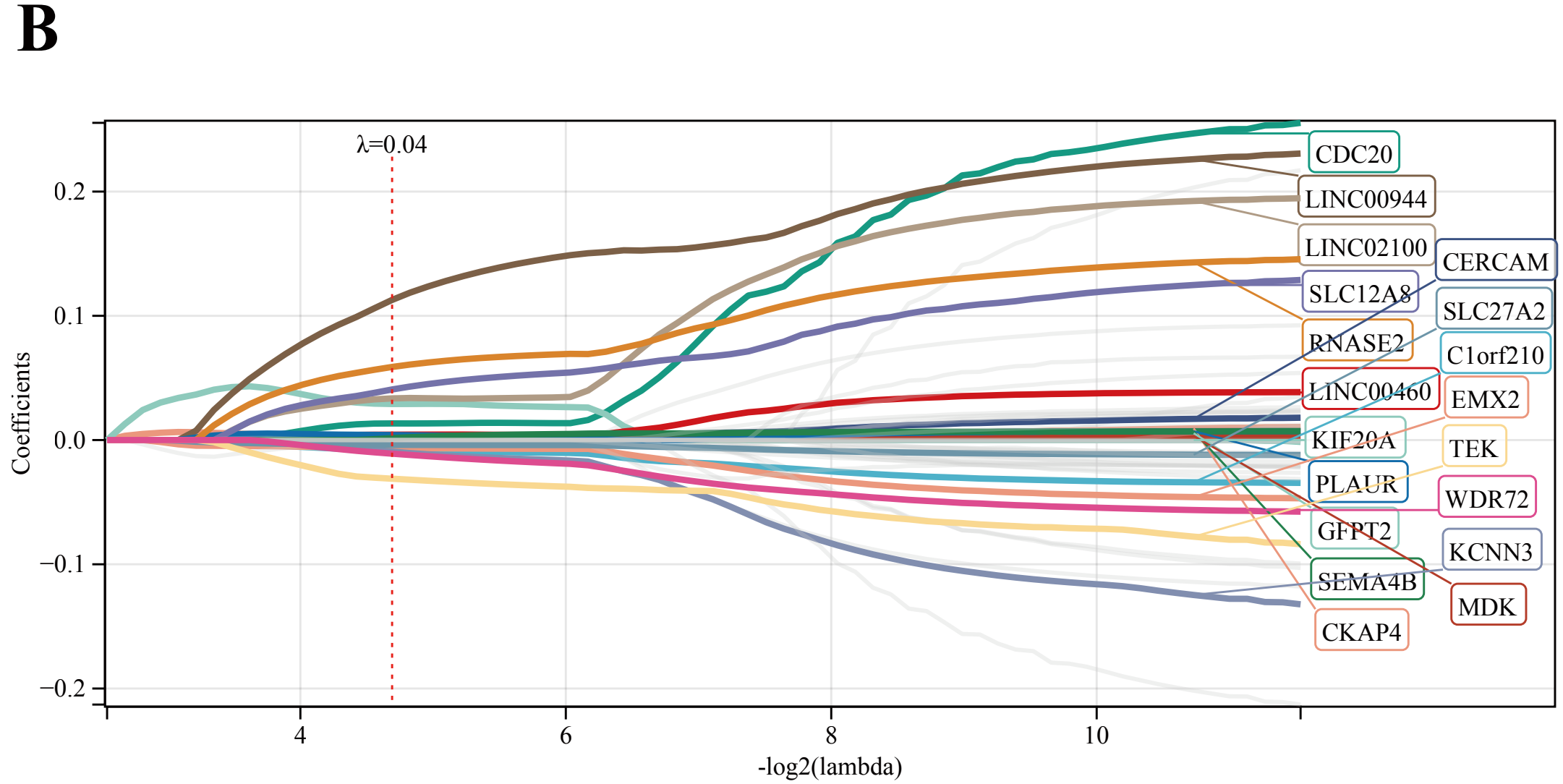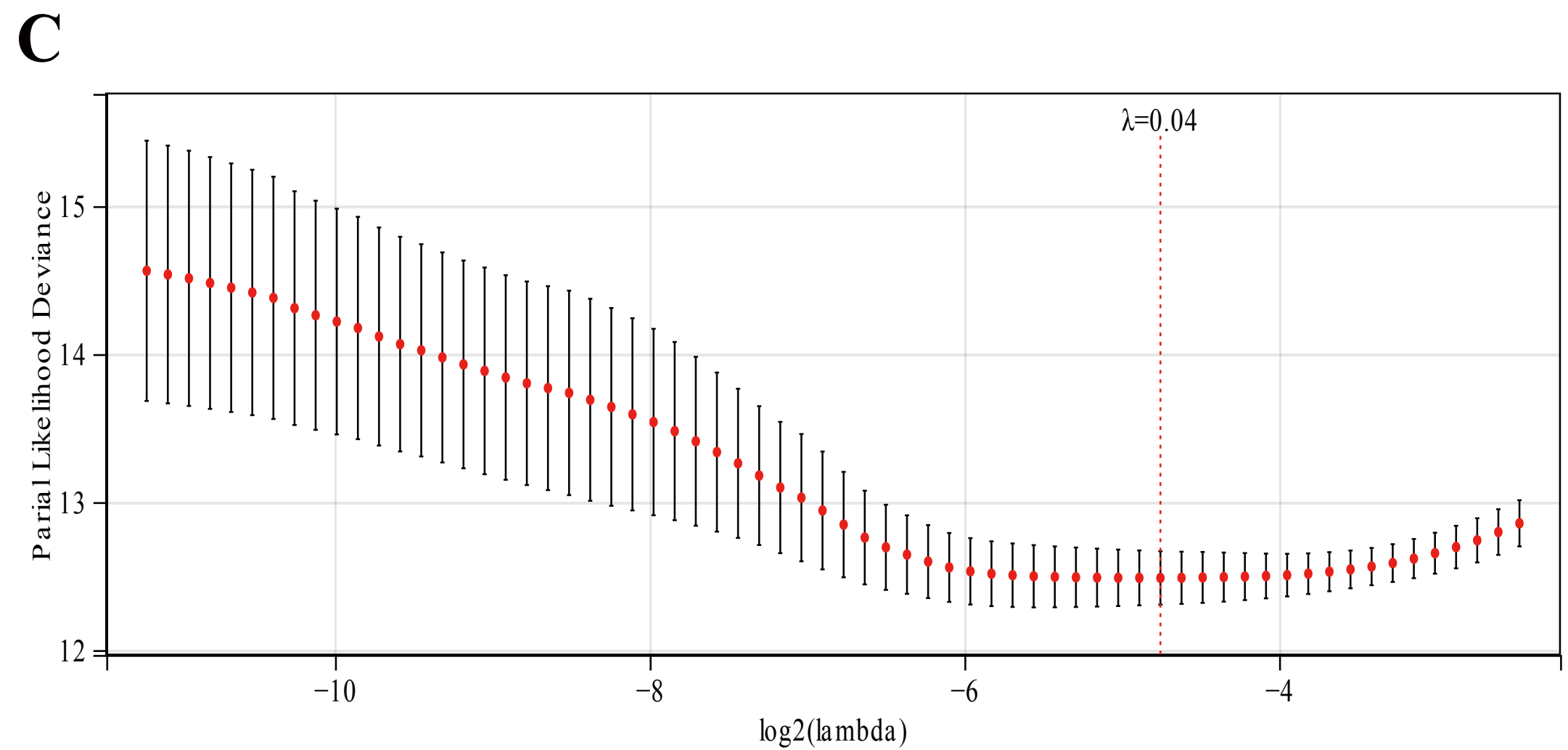

Supplement: Supplementary file 2 — Supporting Information 2 Figure S2: Construction of a MYDGF‐related prognostic gene signature in clear cell renal cell carcinoma. (A) Forest plot showing the hazard ratios (HRs) and 95% confidence intervals (CIs) of the Top 50 MYDGF‐associated genes significantly correlated with patient prognosis based on univariate Cox proportional hazards regression analysis. (B) Least absolute shrinkage and selection operator (LASSO) Cox regression coefficient profiles of candidate MYDGF‐associated genes, illustrating the trajectories of regression coefficients with varying penalty parameters (λ). (C) Cross‐validation plot for LASSO Cox regression showing the partial likelihood deviance as a function of log (λ). The dashed vertical line indicates the optimal value of λ selected by minimum criteria. [file HUMU-2026-5262666-s002.pdf]
